# Postoperative Nausea and Vomiting in the Ambulatory Surgery Center: A Narrative Review

**DOI:** 10.3390/medicines11070016

**Published:** 2024-08-09

**Authors:** Justin Bell, Adam Bindelglass, Jennifer Morrone, Sherwin Park, Ana Costa, Sergio Bergese

**Affiliations:** 1 Department of Anesthesiology, Renaissance School of Medicine, Stony Brook University, Stony Brook, NY 11794, USA; justin.bell@stonybrookmedicine.edu (J.B.); adam.bindelglass@stonybrookmedicine.edu (A.B.); sherwin.park@stonybrookmedicine.edu (S.P.); ana.costa@stonybrookmedicine.edu (A.C.); 2 Renaissance School of Medicine, Stony Brook University, Stony Brook, NY 11794, USA; jennifer.morrone@stonybrookmedicine.edu

**Keywords:** postoperative nausea and vomiting, antiemetic, ambulatory surgery, anesthetic complications, patient satisfaction, cost-effective anesthesia

## Abstract

Postoperative nausea and vomiting (PONV) is a common complication of ambulatory surgery, leading to numerous deleterious effects such as decreased patient satisfaction, prolonged recovery unit stays, and rarely, more serious complications such as aspiration pneumonia or wound dehiscence. In this paper, we present a narrative review of the literature regarding common risk factors for PONV including patient factors, surgical factors, and anesthetic factors. We then will review anesthetic techniques and antiemetic drugs demonstrated to mitigate the risk of PONV. Finally, we discuss the potential economic benefits of PONV prophylaxis in the perioperative ambulatory setting.

## 1. Introduction

Beginning in the early 1980s, ambulatory surgery has comprised a growing proportion of operative procedures likely owing to advances in medicine and changes in payment infrastructure. As opposed to inpatient surgery, where the patient is admitted to the hospital for surgical and/or medical management, ambulatory surgery refers to patients that come to surgery from home and return home after surgery. In 2006, there were approximately 34.7 million ambulatory procedural visits, of which 14.9 million took place in freestanding ambulatory surgery centers [1]. This represented a nearly 300% increase in freestanding ambulatory surgery between 1996 and 2006. According to the agency for healthcare research and quality, 19.2 million outpatient surgical services took place in 2018 and reflected 49% of community hospital revenue [2]. Ambulatory surgery in freestanding centers has been shown to decrease intraoperative time, surgical procedural time, and postoperative anesthesia care unit (PACU) stay times, all of which led to an overall average time reduction from 146.6 min in hospital-based centers to 97.7 min [1]. Advancements in anesthesia practices have led to faster emergence, fewer anesthetic complications, and improved postoperative analgesia: likely a major contributing factor to the liberalization of procedures from hospital-based inpatient settings. Anesthetic complications and unexpected hospital admissions infrequently occur due to the safety of modern anesthesia. However, postoperative nausea and vomiting (PONV) still leads to socioeconomic impacts in both hospital-based and ambulatory surgery centers alike [3]. Some negative effects of PONV include patient discomfort, costly novel antiemetics for PONV prophylaxis or treatment, increased length of PACU stay, and overall reduction in patient satisfaction. PONV may also lead to more serious complications including pulmonary aspiration or suture dehiscence. While anesthesiologists should mitigate the consequences of PONV in all care settings, it is of utmost importance in ambulatory care settings which are designed to minimize time from operating room to discharge while maintaining national and institutional quality care standards.

## 2. Risk Factors

There are many established risk factors for PONV. The strongest risk factors can be divided into those that are non-modifiable (female sex, age, type and duration of surgery, history of PONV, and nonsmoking) and those that are modifiable (use of volatile anesthetic, general vs. regional, and use of postoperative opioids [4]. With the fast time to discharge of ambulatory surgery, there is growing awareness of a separate metric: post-discharge nausea and vomiting (PDNV). PDNV is estimated to occur in 37% of same day surgery patients and can be more challenging to treat without intravenous (IV) access [5]. A retrospective cohort study of 2170 ambulatory surgery patients in the United States from 2007 to 2008 found female gender, age less than 50, history of PONV, opioid administration in PACU, and nausea in PACU to be independent risk factors for PDNV with an incidence of 7%, 20%, 28%, 53%, 60%, and 89% as these factors accumulate. In addition, providing appropriate prophylaxis for PONV is financially beneficial to an institution. A retrospective study of 56,523 surgical patients at a single hospital over two years found that PONV prophylaxis administration was economically beneficial to administer when compared to the costs of treating patients returning to the hospital with sustained PDNV [6].

## 3. Prevention

Although complete prevention of PONV may be difficult in certain cases, PONV prophylaxis starts with reducing risk via the mitigation of modifiable risk factors. A multimodal pain management regimen that minimizes opioid consumption should be utilized. Perioperative acetaminophen, NSAIDs, and COX 2 inhibitors have all been shown in randomized controlled trials to decrease postoperative opioid consumption and PONV [7]. The use of peripheral nerve blocks has also been demonstrated to reduce opiate requirements and PONV. Interestingly, a meta-analysis of randomized controlled trials found that while spinal anesthesia also decreased opiate consumption, it did not decrease PONV and was associated with a 35 min increased total ambulatory surgical center time [8]. Epidural analgesia has proven beneficial; however, it is not usually applicable to the ambulatory care setting. The use of propofol TIVA is comparable to the benefit of a volatile anesthetic plus one antiemetic prophylaxis in terms of PONV risk reduction [9]. Sub-hypnotic propofol infusions mixed with volatile anesthesia have been shown to significantly reduce PONV as well. However, propofol infusions are typically more costly than low-flow volatile gas anesthesia and may prolong emergence times depending on the dosage and/or duration of the infusion, outweighing the financial benefit of preventing PONV when compared to quicker-to-emerge anesthetics and equally efficacious PONV prophylaxis. There is a diminishing return to preventing PONV with propofol infusions depending on institutional drug pricing agreements. The reversal of a neuromuscular blockade with sugammadex vs. neostigmine and glycopyrrolate presents a similar scenario. A large retrospective cohort study of 10,912 patients found that reversal with sugammadex resulted in a 2% absolute reduction in PONV rates [10]. However, the significant cost of sugammadex probably outweighs the financial benefit of its use in PONV reduction.

In addition to minimizing PONV risk by mitigating the modifiable risk factors, prophylactic therapy should be administered during the perioperative period. The number of prophylactic agents administered depends on the number of PONV risk factors. For patients with 1–2 risk factors, 2 agents should be given. For patients with more than two risk factors, three or more agents should be given [4].

## 4. Prophylaxis

Prophylaxis is paramount in the prevention of PONV. Various classes of medications serve as prophylactic agents such as serotonin type 3 (5-HT3) receptor antagonists, corticosteroids, antimuscarinics, neurokinin-1 (NK-1) receptor antagonists, dopamine 2 (D2) antagonists, and combination drugs. Below we review the recommended medications in each of these classes of prophylactic agents.

### 4.1. Serotonin Type 3 Receptor Antagonists

#### 4.1.1. Ondansetron

Serotonin type 3 (5-HT3) receptor antagonists are the most widely used class of antiemetic in the post-anesthesia setting, with ondansetron being considered the “gold standard” antiemetic. Ondansetron has a number needed to treat (NNT) of six for vomiting and seven for nausea [11]. Ondansetron is typically dosed at 4 mg IV, which is equivalent to the 8 mg orally disintegrating tablet. Studies have found 4 mg IV ondansetron to be the most effective at decreasing postoperative nausea, vomiting, and the need for rescue treatment when given 30 min prior to emergence from anesthesia [12,13]. A large meta-analysis involving 10,390 adults and 1688 children from 48 trials found that 8 mg IV ondansetron was not found to be superior to 4 mg IV ondansetron in preventing PONV. However, both the 4 mg and 8 mg doses were superior to 1 mg IV [14]. The most common side effects associated with ondansetron are headache, dry mouth, constipation and, at higher doses, the potential for QT prolongation [15]. A randomized controlled trial (RCT) among patients undergoing outpatient surgery found that ondansetron is an ideal choice for PONV prevention in the ambulatory setting, as it significantly lowered nausea scores during the initial 2 h postoperative observation period and did not cause significant sedation to preclude discharge [16].

#### 4.1.2. Ramosetron

Ramosetron is a selective 5-HT3 antagonist that is more potent and has longer antiemetic effects than older 5-HT3 antagonists. It is typically dosed at a single dose of 0.3 mg IV for adults. A meta-analysis found 0.3 mg of ramosetron and 3 mg of granisetron to have similar efficacy in preventing PONV [17]. Recent studies have also found ramosetron to be more effective at preventing PONV than ondansetron. In a RCT, ramosetron was significantly more efficient than ondansetron at 0–6, 0–24, and 6–24 h, but not after 24–48 h [18]. In addition, this study found ramosetron to be associated with fewer side effects, such as headache, dizziness, and drowsiness, when compared to ondansetron. Furthermore, a large meta-analysis showed that ramosetron was more effective for postoperative nausea than aprepitant, ondansetron, and placebo, with a 69.2% probability of being the best prophylaxis for postoperative nausea during the first 24 h [19]. Of note, this trial was mostly for patients undergoing inpatient neurosurgery which may confound its effectiveness in the outpatient environment.

#### 4.1.3. Palonosetron

Palonosetron is another long-acting 5-HT3 antagonist with a half-life of approximately 40 h and antiemetic effects lasting into the second and third postoperative day [20]. These properties of palonosetron may provide additional benefits, but there is conflicting evidence on its efficacy. A meta-analysis found palonosetron to be superior in the prevention of PONV when compared to ondansetron, ramosetron, and granisetron [18]. A RCT found a dose-dependent decrease in PONV up to day 3 for 0.5, 1, and 1.5 mcg/kg palonosetron doses [21]. However, another RCT comparing palonosetron to ondansetron found a lower incidence of PONV in the palonosetron group only at time 2–24 h, with no difference for 0–2 h or 24–28 h [20]. Both RCT studies were small and the impact of longer-acting 5-HT3 agonists merits further investigation.

### 4.2. Corticosteroids

Corticosteroids such as dexamethasone are also potent antiemetics when given post-induction, with a NNT of seven for vomiting at a dose of 8 mg, and a comparable incidence of PONV when compared to 5-HT3 antagonists [22,23]. Corticosteroids target intracellular receptors in the nucleus tractus solitarius; therefore, most of its physiological effects may take several hours to manifest. For this reason, corticosteroids have been shown to have a stronger effect on the reduction in nausea and vomiting in the late postoperative stage (up to 24 h). Its prolonged half-life (36–72 h) may also be responsible for this delayed antiemetic effect [22]. In a RCT composed of women receiving dexamethasone vs. ondansetron undergoing gynecologic surgery, the dexamethasone group showed an advantage over the ondansetron group in rates of vomiting only at the 24 h mark [24]. Further, following ambulatory same-day gynecologic surgery, dexamethasone had no effect in preventing PONV in the first 0–3 h postoperatively and showed efficacy only after 6 h [25]. These findings may suggest a decreased efficacy of corticosteroids in the ambulatory setting. Yet, a large meta-analysis found that dexamethasone and 5-HT3 antagonists performed equivalently in both the immediate postoperative period and up to 24 h after surgery [26].

Despite some concerns over the side effects of corticosteroid use, many studies have shown that a single dose of dexamethasone has no effect on wound healing and causes only a minimal rise in blood glucose levels—even among diabetics [27]. With regard to dosing, a meta-analysis of 60 RCTs comparing dexamethasone dosing of 4 mg vs. 8 mg found no significant difference in PONV between the two doses [23]. However, higher doses of dexamethasone have been shown to result in decreased postoperative pain and decreased opioid consumption at doses of 0.11 to 0.2 mg/kg [28], which may provide a secondary benefit of reduction of other side effects of opioid administration. Furthermore, dexamethasone reduced the analgesic need compared to 5-HT3 antagonists (MH-odds of 0.64), offering the advantage of decreased opioid requirements postoperatively [26].

### 4.3. Antimuscarinics

Scopolamine is a centrally acting antimuscarinic agent initially developed to prevent motion sickness but was approved by the United States Food and Drug Administration (FDA) for the prevention of PONV in 2001. Compared with other administration routes, the sustained-release transdermal scopolamine (TDS) patch provides long-acting activity at much lower quantities (1.5 mg delivered at 5 µg per hour for ~72 h). This long-acting duration has proven to be of particular benefit in the outpatient surgical setting, as TDS can provide sustained relief of PONV after discharge [29]. Several adverse events related to its anticholinergic properties, such as sedation, dry mouth, blurred vision, and confusion can occur, but are more prevalent with IV scopolamine use than steady release from the transdermal patch [30]. In a systematic review and meta-analysis, TDS was associated with a significantly reduced risk for postoperative nausea (RR = 0.59), postoperative vomiting (RR = 0.68), and PONV (RR = 0.73) for the first 24 h following the start of anesthesia [31]. It was found to have similar efficacy when given the night before versus on the day of surgery. In addition, a RCT found the combined use of TDS and ondansetron had lower rates of nausea and vomiting and the use of rescue medication used 24 h after ambulatory surgery compared with ondansetron only patients (48% vs. 39%, *p* = 0.021) [32].

### 4.4. Neurokinin-1 Receptor Antagonists

The neurokinin-1 (NK-1) receptor is expressed in vagal afferents and brain areas involved in the vomiting reflex, including the tractus solitarius and area postrema. NK-1 antagonists suppress interaction with its natural ligand, substance P, thereby providing antiemetic activity in the central nervous system. NK-1 antagonists such as aprepitant, casopitant, and rolapitant have been developed as antiemetic agents. Aprepitant, the first in this medication class to be approved by the FDA for the management of PONV, is a highly selective NK-1 antagonist administered orally with a 9 to 14 h half-life. Aprepitant was found to be effective when given orally 1–3 h prior to surgery [33]. Fosaprepitant is a water-soluble prodrug of aprepitant that is given intravenously, rapidly transforming into aprepitant within 30 min [34]. NK-1 antagonists have few reported side effects, with the most frequently reported adverse events being headache (2.5–22%), dizziness (7.5–19%), and constipation (7.2–9%) [35].

A recent meta-analysis of 41 single drugs and 51 drug combinations found NK-1 antagonists to be the most effective drug class at preventing postoperative vomiting, and single NK-1 antagonists (fosaprepitant, casopitant, aprepitant) were as effective as most of the drug combinations. Importantly, NK-1 antagonists were found to be much more effective at preventing vomiting than nausea [36]. A large meta-analysis found fosaprepitant (130 mg) to be the most effective prophylactic medication for postoperative vomiting, both between 0–24 and 0–48 h postoperatively, compared to other antiemetics including ondansetron [19]. Another study found all dosages of aprepitant (40, 80, and 125 mg) were effective in reducing the incidence of postoperative vomiting, but not the rates of nausea [35]. However, when given in combination with other drugs, NK-1 antagonists can be highly effective at preventing both nausea and vomiting postoperatively. Treatment effects for combination aprepitant–palonosetron had a relative ratio (RR) as low as 0.01 (0.00 to 0.19) [36]. Another RCT found that supplementation of 80 mg aprepitant in combination with dexamethasone and ondansetron substantially improved the effects of PONV (OR: 0.36; 95% CI: 0.16, 0.82; *p* = 0.01) [33].

### 4.5. Dopamine 2 Antagonists

Dopamine antagonists, specifically targeted against subtype D2 receptors located in the chemoreceptor trigger zone, have been one of the first-line drug choices to treat PONV. Although older D2 antagonists such as droperidol have fallen out of favor due to QT prolongation and concern for cardiac arrhythmias, selective D2/D3 antagonists such as amisulpride have a low tendency to cause side effects [37]. A meta-analysis found Qtc prolongation among amisulpride use to be dose-dependent, with a single IV dose of 5 mg or 10 mg being safe and effective for the prevention of PONV [37]. At 5 mg, the incidence of PONV, vomiting, nausea, and rescue medication use were all reduced compared to placebo, with the 1 and 20 mg doses appearing to be less effective, suggesting a bell-shaped dose-response [38]. Recent studies have also shown that amisulpride given at the time of anesthesia induction, in combination with a standard antiemetic, such as ondansetron, significantly reduced PONV in patients undergoing a wide range of operations [39]. Amisulpride has many attractive features, including a low propensity for drug interactions and a rapid onset of action, making it greatly beneficial in the ambulatory surgery setting [38].

### 4.6. Combination Drugs

In current anesthesia practice, these aforementioned drugs are typically given in combination to optimize antiemetic effects. There is robust evidence to show that combinations of antiemetics are more effective than single drugs in preventing PONV [36]. Further, a meta-analysis of 14 pooled RCTs found that dexamethasone combined with other antiemetics (including ondansetron, metoclopramide, granisetron, and palonosetron) was more effective than these single antiemetics alone in preventing PONV in both the early and late postoperative period [40]. Most commonly, dexamethasone 4 or 8 mg is combined with a 5-HT3 receptor antagonist [4]. Notably, palonosetron plus dexamethasone had lower rates of PONV than ondansetron plus dexamethasone [41]. Recent studies have also begun looking at the efficacy of triple-agent and even quadruple-agent combinations for high-risk patients [42]. One RCT found the combination of aprepitant 80 mg, dexamethasone 4–8 mg, and ondansetron 4 mg was superior to dexamethasone and ondansetron alone in preventing PONV after laparoscopic surgery [43]. However, more studies are needed to clarify the dosage, administration time and efficacy of using more than two agents in PONV prophylaxis.

## 5. Cost-Effective Ambulatory Practice

The economic impact of PONV on outpatient surgical centers has been an area of close attention for a considerable time. In 1994, it was estimated that the financial cost of additional care, resources, and lost revenue related to PONV was USD 437.06 per patient [44]. In a retrospective study conducted on operative cases from 2005 to 2007, it was estimated that the charges incurred by patients in relation to PONV were approximately USD 3.66 per antiemetic dose and the hospital cost was USD 0.304 per antiemetic dose [6]. Therefore, based on studies showing the benefit of PONV prophylaxis described above and not accounting for inflation, the cost estimate of prophylaxis to a high-risk patient is approximately USD 10.98 and USD 0.912 to the hospital. In 2000, Gan et al. reported that patients were willing to pay between USD 56 and USD 100 to prevent PONV [45]. Further, Diez showed that parents were willing to pay about GBP 50 to prevent PONV in 1998, which was equivalent to roughly USD 80 at the time [46]. These studies suggest that patients are willing to pay far more than the cost incurred to prevent PONV when using a risk-guided approach. The data reported by Dzwonczyk et al. suggest that hospital profit would be increased by approximately USD 140,866 over the course of a 2-year period if all surgical patients were prophylactically treated. This number decreased to USD 105,650 when reduced to only patients with three risk factors [6]. However, when determining the economic impact of PONV prophylaxis in ambulatory centers, it is important to realize that, on average, procedures take about 31.8 min longer when performed in a hospital setting versus an ambulatory center according to Munnich et al. [47]. This reduction in time to discharge could generate savings ranging from USD 364 to USD 1000 per ambulatory surgical center case under standard circumstances [47]. According to Parra-Sanchez et al., patients with PONV spent one hour longer in the PACU and incurred a USD 75 incremental cost per patient [48]. These data further demonstrates the reduction in efficiency and consequently profit that PONV imposes on ambulatory centers. Waddle et al. estimated that the average cost of PACU care after the first half hour was USD 55.22 per half hour, a number that would represent approximately USD 104 in 2023 [49]. When using the estimated extra hour prolongation in PACU stay times for patients with PONV, this would suggest a cost of approximately USD 208 for each patient with PONV. Ultimately, it is readily apparent that a reduction in PONV can result in large profit savings for ambulatory centers and hospitals alike.

## 6. Discussion

The impact of PONV on the patient’s experience and hospital profits demonstrates the need for proactivity by the perioperative team. It is estimated that the incidence of PONV is 37% throughout the general population, a statistic that drastically increases to a maximum of 80% when risk stratifying using the Apfel risk predictors [4]. In addition to a relatively high incidence, patients frequently report nausea and vomiting as one of the most undesirable outcomes of anesthesia [49]. In a 1999 study, patients were asked to divide USD 100 amongst outcomes they wished to avoid. The top five undesirable outcomes, from most to least undesirable, were vomiting, gagging on the endotracheal tube, pain, nausea, and recall without pain [50].

As described above, there are multiple medications and modifiable risk factors that can help reduce the overall risk of PONV. It is standard practice in many institutions to utilize a combination approach to most, if not all, patients. Standard combinations of 5-HT3 receptor antagonists and dexamethasone have been shown to be superior to single therapy alone [51]. The supporting evidence for other combination therapies including dexamethasone and aprepitant, aprepitant and ondansetron, and midazolam plus standard antiemetics is also robust, further demonstrating the superiority of combination therapy [4]. Newer studies have begun to examine the efficacy of triple or quadruple therapy for PONV prophylaxis. Dexamethasone, ondansetron, and modifiable risk factor reduction was compared to the same regimen plus aprepitant in high risk (Apfel 3 or 4) patients and was determined to significantly reduce PONV [43]. Based on the currently available research, guidelines therefore recommend two agents in low-risk patients and three or more agents in high-risk patients. Given the promising results from several large studies, it would be beneficial to further examine combination therapies, especially triple and quadruple therapy, and their relative risk reduction in both low- and high-risk populations. This review focuses on pharmacological approaches to the prophylaxis and treatment of PONV. Non-pharmacological approaches have included acupuncture, aromatherapy, transcutaneous electrical stimulation and relaxation techniques, among others.

A notable limitation of this narrative review is that much PONV research is conducted at academic university hospitals. While we tried to include studies focusing on the ambulatory environment, many large RCTs we included were conducted outside the ambulatory environment. Differences in anesthetic duration and practice habits at ambulatory centers vs. academic hospitals are a confounding variable. More RCTs conducted at ambulatory centers are needed.

Prophylactic pharmacologic interventions may initially appear to be a significant expense to both the healthcare system and the patient. However, various studies have shown clear revenue savings, largely through decreased length of stay. In addition, patient retention and satisfaction would trump the marginal medication costs for PONV prophylaxis. The cost to the individual patient of regularly utilized antiemetic medications is far less than what the average patient would be willing to pay to prevent PONV. Appropriate risk stratification, institutional protocols, and application of combination therapy for high-risk groups could help to decrease unnecessary spending for low-risk patients and potentially increase revenue. Further studies should aim to examine the NNT and risk reduction in novel combination therapies and apply this information to devise the most cost-effective PONV strategies without compromising clinical care. It is likely that applying the same combination therapies described above to ambulatory centers would similarly improve patient satisfaction and reduce length of stay. However, further studies should be conducted to specifically examine cost-savings and patient satisfaction in the growing number of ambulatory surgery centers.

## Data Availability

No new data were created or analyzed in this study. Data sharing is not applicable to this article.

## References

[1] Cullen K.A., Hall M.J., Golosinskiy A. (2009). Ambulatory surgery in the United States, 2006. Natl. Health Stat. Rep..

[2] McDermott K.W., Liang L. (2021). Overview of Operating Room Procedures During Inpatient Stays in U.S. Hospitals, 2018. Healthcare Cost and Utilization Project (HCUP) Statistical Briefs.

[3] Gress K., Urits I., Viswanath O., Urman R.D. (2020). Clinical and economic burden of postoperative nausea and vomiting: Analysis of existing cost data. Best Pract. Res. Clin. Anaesthesiol..

[4] Gan T.J., Belani K.G., Bergese S., Chung F., Diemunsch P., Habib A.S., Jin Z., Kovac A.L., Meyer T.A., Urman R.D. (2020). Fourth Consensus Guidelines for the Management of Postoperative Nausea and Vomiting. Anesth. Analg..

[5] Apfel C.C., Philip B.K., Cakmakkaya O.S., Shilling A., Shi Y.Y., Leslie J.B., Allard M., Turan A., Windle P., Odom-Forren J. (2012). Who is at risk for postdischarge nausea and vomiting after ambulatory surgery?. Anesthesiology.

[6] Dzwonczyk R., Weaver T.E., Puente E.G., Bergese S.D. (2012). Postoperative Nausea and Vomiting Prophylaxis from an Economic Point of View. Am. J. Ther..

[7] Marret E., Kurdi O., Zufferey P., Bonnet F. (2005). Effects of nonsteroidal antiinflammatory drugs on patient-controlled analgesia morphine side effects: Meta-analysis of randomized controlled trials. Anesthesiology.

[8] Liu S.S., Strodtbeck W.M., Richman J.M., Wu C.L. (2005). A comparison of regional versus general anesthesia for ambulatory anesthesia: A meta-analysis of randomized controlled trials. Anesth. Analg..

[9] Uchinami Y., Takikawa S., Takashima F., Maeda Y., Nasu S., Ito A., Saito T. (2019). Incidence of postoperative nausea and vomiting is not increased by combination of low concentration sevoflurane and propofol compared with propofol alone in patients undergoing laparoscopic gynecological surgery. JA Clin. Rep..

[10] Ju J.-W., Hwang I.E., Cho H.-Y., Yang S.M., Kim W.H., Lee H.-J. (2023). Effects of sugammadex versus neostigmine on postoperative nausea and vomiting after general anesthesia in adult patients:a single-center retrospective study. Sci. Rep..

[11] Tramèr M.R., Reynolds D.J., Moore R.A., McQuay H.J. (1997). Efficacy, dose-response, and safety of ondansetron in prevention of postoperative nausea and vomiting: A quantitative systematic review of randomized placebo-controlled trials. Anesthesiology.

[12] Hartsell T., Long D., Kirsch J.R. (2005). The efficacy of postoperative ondansetron (Zofran) orally disintegrating tablets for preventing nausea and vomiting after acoustic neuroma surgery. Anesth. Analg..

[13] Cruz N.I., Portilla P., Vela R.E. (2008). Timing of ondansetron administration to prevent postoperative nausea and vomiting. P. R. Health Sci. J..

[14] Figueredo E.D., Canosa L.G. (1998). Ondansetron in the prophylaxis of postoperative vomiting: A meta-analysis. J. Clin. Anesth..

[15] Singh K., Jain A., Panchal I., Madan H., Gupta A., Sharma A., Gupta S., Kostojchin A., Singh A., Sandhu I.S. (2023). Ondansetron-induced QT prolongation among various age groups: A systematic review and meta-analysis. Egypt. Heart J..

[16] Scuderi P., Wetchler B., Sung Y.F., Mingus M., DuPen S., Claybon L., Leslie J., Talke P., Apfelbaum J., Sharifi-Azad S. (1993). Treatment of postoperative nausea and vomiting after outpatient surgery with the 5-HT3 antagonist ondansetron. Anesthesiology.

[17] Kim W.O., Koo B.N., Kim Y.K., Kil H.K. (2011). Ramosetron for the prevention of postoperative nausea and vomiting (PONV): A meta-analysis. Korean J. Anesth..

[18] Gao C., Li B., Xu L., Lv F., Cao G., Wang H., Wang F., Wu G. (2015). Efficacy and safety of ramosetron versus ondansetron for postoperative nausea and vomiting after general anesthesia: A meta-analysis of randomized clinical trials. Drug Des. Dev. Ther..

[19] Chen Y., Chang J. (2020). Anti-emetic Drugs for Prophylaxis of Postoperative Nausea and Vomiting After Craniotomy: An Updated Systematic Review and Network Meta-Analysis. Front. Med..

[20] Balyan R., Kumar S., Lalitha K., Aneja S., George J.S. (2022). A Comparative Study of Palonosetron with Ondansetron for Prophylaxis of Postoperative Nausea and Vomiting (PONV) Following Laparoscopic Gynaecological Surgeries. Rom. J. Anaesth. Intensive Care.

[21] Bicer C., Aksu R., Ulgey A., Madenoglu H., Dogan H., Yildiz K., Boyaci A. (2011). Different doses of palonosetron for the prevention of postoperative nausea and vomiting in children undergoing strabismus surgery. Drugs R&D.

[22] Henzi I., Walder B., Tramèr M.R. (2000). Dexamethasone for the prevention of postoperative nausea and vomiting: A quantitative systematic review. Anesth. Analg..

[23] De Oliveira G.S., Castro-Alves L.J., Ahmad S., Kendall M.C., McCarthy R.J. (2013). Dexamethasone to prevent postoperative nausea and vomiting: An updated meta-analysis of randomized controlled trials. Anesth. Analg..

[24] Abdulhussain A.S. (2022). Combination of dexamethasone and ondansetron in prophylaxis nausea and vomiting in gynecological operation. J. Popul. Ther. Clin. Pharmacol..

[25] Yuksek M.S., Alici H.A., Erdem A.F., Cesur M. (2003). Comparison of prophylactic anti-emetic effects of ondansetron and dexamethasone in women undergoing day-case gynaecological laparoscopic surgery. J. Int. Med. Res..

[26] Singh P.M., Borle A., Panwar R., Makkar J.K., McGrath I., Trikha A., Sinha A. (2018). Perioperative antiemetic efficacy of dexamethasone versus 5-HT3 receptor antagonists: A meta-analysis and trial sequential analysis of randomized controlled trials. Eur. J. Clin..

[27] Pang Q.Y., Wang J.Y., Liang X.L., Jiang Y., Liu H.L. (2023). The safety of perioperative dexamethasone with antiemetic dosage in surgical patients with diabetes mellitus: A systematic review and meta-analysis. Perioper. Med..

[28] De Oliveira G.S., Almeida M.D., Benzon H.T., McCarthy R.J. (2011). Perioperative single dose systemic dexamethasone for postoperative pain: A meta-analysis of randomized controlled trials. Anesthesiology.

[29] Sah N., Ramesh V., Kaul B., Dalby P., Shestak K., Vallejo M.C. (2009). Transdermal scopolamine patch in addition to ondansetron for postoperative nausea and vomiting prophylaxis in patients undergoing ambulatory cosmetic surgery. J. Clin. Anesth..

[30] Antor M.A., Uribe A.A., Erminy-Falcon N., Werner J.G., Candiotti K.A., Pergolizzi J.V., Bergese S.D. (2014). The effect of transdermal scopolamine for the prevention of postoperative nausea and vomiting. Front. Pharmacol..

[31] Apfel C.C., Zhang K., George E., Shi S., Jalota L., Hornuss C., Fero K.E., Heidrich F., Pergolizzi J.V., Cakmakkaya O.S. (2010). Transdermal scopolamine for the prevention of postoperative nausea and vomiting: A systematic review and meta-analysis. Clin. Ther..

[32] Gan T.J., Sinha A.C., Kovac A.L., Jones R.K., Cohen S.A., Battikha J.P., Deutsch J.S., Pergolizzi J.V., Glass P.S. (2009). A randomized, double-blind, multicenter trial comparing transdermal scopolamine plus ondansetron to ondansetron alone for the prevention of postoperative nausea and vomiting in the outpatient setting. Anesth. Analg..

[33] Liu Y., Chen X., Wang X., Zhong H., He H., Liu Y., Liao Y., Pan Z., Hu W., Liu W. (2023). The efficacy of aprepitant for the prevention of postoperative nausea and vomiting: A meta-analysis. Medicine.

[34] Lasseter K.C., Gambale J., Jin B., Bergman A., Constanzer M., Dru J., Han T.H., Majumdar A., Evans J.K., Murphy M.G. (2007). Tolerability of fosaprepitant and bioequivalency to aprepitant in healthy subjects. J. Clin. Pharmacol..

[35] Liu M., Zhang H., Du B.X., Xu F.Y., Zou Z., Sui B., Shi X.Y. (2015). Neurokinin-1 receptor antagonists in preventing postoperative nausea and vomiting: A systematic review and meta-analysis. Medicine.

[36] Weibel S., Pace N.L., Schaefer M.S., Raj D., Schlesinger T., Meybohm P., Kienbaum P., Eberhart L.H.J., Kranke P. (2021). Drugs for preventing postoperative nausea and vomiting in adults after general anesthesia: An abridged Cochrane network meta-analysis. J. Evid. Based Med..

[37] Zhang L.F., Zhang C.F., Tang W.X., He L., Liu Y., Tian D.D., Ai Y.Q. (2020). Efficacy of amisulpride on postoperative nausea and vomiting: A systematic review and meta-analysis. Eur. J. Clin. Pharmacol..

[38] Kranke P., Eberhart L., Motsch J., Chassard D., Wallenborn J., Diemunsch P., Liu N., Keh D., Bouaziz H., Bergis M. (2013). I.V. APD421 (amisulpride) prevents postoperative nausea and vomiting: A randomized, double-blind, placebo-controlled, multicentre trial. Br. J. Anaesth..

[39] Kranke P., Bergese S.D., Minkowitz H.S., Melson T.I., Leiman D.G., Candiotti K.A., Liu N., Eberhart L., Habib A.S., Wallenborn J. (2018). Amisulpride Prevents Postoperative Nausea and Vomiting in Patients at High Risk: A Randomized, Double-blind, Placebo-controlled Trial. Anesthesiology.

[40] Awad K., Ahmed H., Abushouk A.I., Al Nahrawi S., Elsherbeny M.Y., Mustafa S.M., Attia A. (2016). Dexamethasone combined with other antiemetics versus single antiemetics for prevention of postoperative nausea and vomiting after laparoscopic cholecystectomy: An updated systematic review and meta-analysis. Int. J. Surg..

[41] Sharma A.N., Shankaranarayana P. (2015). Postoperative Nausea and Vomiting: Palonosetron with Dexamethasone vs. Ondansetron with Dexamethasone in Laparoscopic Hysterectomies. Oman Med. J..

[42] Ziemann-Gimmel P., Goldfarb A.A., Koppman J., Marema R.T. (2014). Opioid-free total intravenous anaesthesia reduces postoperative nausea and vomiting in bariatric surgery beyond triple prophylaxis. Br. J. Anaesth..

[43] De Morais L.C., Sousa A.M., Flora G.F., Grigio T.R., Guimarães G.M.N., Ashmawi H.A. (2018). Aprepitant as a fourth antiemetic prophylactic strategy in high-risk patients: A double-blind, randomized trial. Acta Anaesthesiol. Scand..

[44] Carroll N.V., Miederhoff P.A., Cox F.M., Hirsch J.D. (1994). Hirsch. Costs Incurred by outpatient surgical centers in managing postoperative nausea and vomiting. J. Clin. Anesth..

[45] Gan T.J., Sloan F., de L Dear G., El-Moalem H.E., Lubarsky D.A. (2000). How Much are Patients Willing to Pay to Avoid Postoperative Nausea and Vomiting. Anesth. Analg..

[46] Diez L. (1998). Assessing the Willingness of Parents to Pay for Reducing Postoperative Emesis in Children. PharmacoEconomics.

[47] Munnich E.L., Parente S.T. (2014). Procedures Take Less Time at Ambulatory Surgery Centers, Keeping Costs Down And Ability to Meet Demand Up. Health Aff..

[48] Parra-Sanchez I., Abdallah R., You J., Fu A.Z., Grady M., Cummings K., Apfel C., Sessler D.I. (2012). A time-motion economic analysis of postoperative nausea and vomiting in ambulatory surgery. Can. J. Anaesth..

[49] Waddle J.P., Evers A.S., Piccirillo J.F. (1998). Postanesthesia Care Unit Length of Stay Quantifying and Assessing Dependent Factors. Anesth. Analg..

[50] Macario A.M., Weinger M.M., Carney S.B., Kim A.B. (1999). Which Clinical Anesthesia Outcomes Are Important to Avoid? The Perspective of Patients. Anesth. Analg..

[51] Som A., Bhattacharjee S., Maitra S., Arora M.K., Baidya D.K. (2016). Combination of 5-HT3 Antagonist and Dexamethasone Is Superior to 5-HT3 Antagonist Alone for PONV Prophylaxis after Laparoscopic Surgeries: A Meta-analysis. Anesth. Analg..

